# Screening of *Saccharomyces* and Non-*Saccharomyces* Wine Yeasts for Their Decarboxylase Activity of Amino Acids

**DOI:** 10.3390/foods11223587

**Published:** 2022-11-11

**Authors:** Gabriella Siesto, Maria Rosaria Corbo, Rocchina Pietrafesa, Milena Sinigaglia, Patrizia Romano, Antonio Bevilacqua

**Affiliations:** 1 School of Agricultural, Forestry, Food and Environmental Sciences, University of Basilicata, Via dell’Ateneo Lucano 10, 85100 Potenza, Italy; 2 Department of Agriculture, Food, Natural Resources, and Engineering, University of Foggia, 71122 Foggia, Italy; 3 Faculty of Economy, Universitas Mercatorum, Piazza Mattei, 10, 00186 Rome, Italy

**Keywords:** decarboxylase activity, amino acids, wine yeasts, *Saccharomyces* strains, non-*Saccharomyces* strains, yeast selection program, consumer health

## Abstract

The type and quantity of precursor amino acids present in grape must that are used by wine yeasts affect the organoleptic and health properties of wine. The aim of this work was to conduct a preliminary screening among *Saccharomyces* and non-*Saccharomyces* indigenous strains, which were previously isolated from different Italian regional grape varieties. This was performed in order to evaluate their decarboxylase activity on certain important amino acids—such as arginine, proline, serine, and tyrosine—that are present in grape must. In particular, a qualitative test on 122 wine yeasts was performed on a decarboxylase medium using arginine, proline, serine, and tyrosine as precursor amino acids. Our results showed a considerable variability among the microbial species tested for this parameter. Indeed, *Saccharomyces cerevisiae* strains exhibited a high decarboxylase capability of the four amino acids tested; moreover, only 10% of the total (i.e., a total of 81) did not show this trait. A high recovery of decarboxylation ability for at least one amino acid was also found for *Zygosaccharomyces bailii* and *Hanseniaspora* spp. These findings can, therefore, promote the inclusion of decarboxylase activity as an additional characteristic in a wine yeast selection program in order to choose starter cultures that possess desirable technological traits; moreover, this also can contribute to the safeguarding of consumer health.

## 1. Introduction

Grape juice composition in addition to the microbiota that conducts the fermentation are the most important factors that affect wine quality [1]. Beyond the properties of the grape, as well as technological factors, the alcoholic fermentation by yeasts represents the key process during the complex course that is the transformation of grape must into wine [2,3]. During wine production, populations of different yeast genera live together and succeed one another [4]. At the early stages of fermentation, low ethanol-tolerant non-*Saccharomyces* species usually are predominant and start the fermentation process, especially yeasts belonging to the genera *Hanseniaspora* [5]. Wine strains of this genus are endowed with the ability to produce fermentation metabolites [6,7] and numerous extracellular enzymes [8], which can affect the quality and aromatic characteristics of wine. The alcohol that progressively accumulates in the medium exerts a selective pressure on the low ethanol-tolerant yeasts; further, they are quickly replaced by *Saccharomyces* wine strains, which, when present, complete the fermentation process [2,9]. The yeast *Saccharomyces cerevisiae* is the main species that is responsible for the fermentation process as it possesses distinctive physiological characteristics when compared to other yeasts, such as the ability to produce and resist high concentrations of ethanol and the tendency to ferment even in the presence of oxygen.

In the middle and final stages of alcoholic fermentation with *S. cerevisiae*, other yeast species—such as *Zygosaccharomyces bailii*—may be present due to their high ethanol tolerance. *Z. bailii* is a well-known food spoilage yeast in the food and beverage industry, due to its ability to resist antimicrobial compounds (such as sulfur dioxide and sorbic acid [10]).

During the fermentation process, in addition to the primary end products (ethanol and CO_2_), yeast metabolism produces many other secondary compounds, thereby influencing the organoleptic profile and its healthy quality. This, therefore, means that wine quality is also a direct consequence of the yeast species/strain that developed during fermentation [5]. As such, the selection of more suitable strains has become the fundamental step that requires attention for a better biotechnological application in winemaking [3]. Further, research groups have addressed these strains’ activities on the wine yeast selection program [11,12] in order to better identify and characterize those starter cultures with desirable oenological features to, therefore, obtain good quality wines [13].

The selection of indigenous yeasts, both of the *Saccharomyces* and non-*Saccharomyces* strains, is based on certain relevant enological traits that are divided into technological and qualitative categories [14,15]. Technological traits are evaluated in order to ensure an efficient fermentative process, such as high fermentation vigor; ethanol tolerance; resistance to sulfur dioxide and heavy metals; good fermentative kinetics at different temperatures; and others [13]. The qualitative characteristics, on the other hand, affect the composition, and therefore the quality, of the finished wine due to the formation (or consumption) of certain compounds or the actions that occur within some of the components of wine. These characteristics concern the demolition and/or transformation of the metabolism components present in the grape must that influence wine composition and quality. In this case, even non-*Saccharomyces* yeasts (although they do not possess some of the important technological qualities) play an important role for enzymatic activities, such as esterases, glycosidases, lipases, proteases, and cellulases [16]. Another relevant enzymatic activity, with a potential impact on the outcome of wine quality, is the decarboxylation of the amino acids present in the must. However, on this topic there is only a small amount of data that is available in the literature.

Amino acids, which can be assimilated by yeasts [17], represent the most important nitrogenous compounds present in grape must. Further, in this regard there is a general trend of arginine and proline being in the highest concentrations [18]. Among the others, serine usually results from glycocol via an enzymatic pathway [19], and tyrosine arises from phenylalanine via 4-hydroxylation [20]. The main metabolism of amino acids determines the production of many aromatic compounds that are important for the final sensory quality of wine [21], which are themselves produced by yeasts during the fermentation process [22]. Another fate of amino acids is found in decarboxylation via exogenous enzymes released by various microorganisms, including yeasts, giving rise to low molecular weight organic compounds (which in high concentrations are detrimental to health [23]). The concentration in wine of these latter compounds is influenced by the abundance of amino acid precursors, the presence of decarboxylase positive microorganisms, and by many oenological parameters—such as alcohol and sulfur dioxide content, pH, and temperature [23,24]—that can increase the concentration of precursor amino acids or favor the growth of positive decarboxylase microorganisms [25].

An extensive body of literature is available on the amino acid decarboxylase activity by lactic acid bacteria in different fermented foods [26,27]. However, there is little literature available that explores the relationships between the content in wine of low-molecular-weight organic compounds and the amino acid decarboxylases of yeasts that are involved in wine production.

The aim of this work was to evaluate *Saccharomyces* and non-*Saccharomyces* indigenous strains. These strains were isolated from different grape varieties and Italian regions for the purposes of analyzing the decarboxylase activity that occurs in certain important amino acids that are present in grape must.

## 2. Materials and Methods

### 2.1. Microorganisms

In this study, we utilized indigenous strains of the species *S. cerevisiae* (81), *Z. bailii* (21), and also of the genus *Hanseniaspora* (20), which came to a total of 122 yeasts. The indigenous strains, belonging to the Yeast Collection of the University of Basilicata (UBYC), were previously isolated from different grape varieties in different Italian regions (Table 1). Yeasts were stored at −20 °C in YPG broth (10 g/L yeast extract; 20 g/L bacteriological peptone; and 20 g/L glucose), then 50% glycerol (Sigma, St. Louis, MO 63304, USA) was added as a protective agent. The strains were grown in a YPG agar medium (bacteriological peptone, 20 g/L; yeast extract, 10 g/L; glucose, 20 g/L; and agar, 15 g/L) at 26 °C for 24 h, before the decarboxylation tests were conducted. All ingredients were purchased from Oxoid (Hampshire, UK).

### 2.2. Amino Acid Decarboxylation Test

The decarboxylation tests were carried out in a modified YPG (bacteriological peptone, 5 g/L; yeast extract, 5 g/L; glucose, 1 g/L; and agar, 15 g/L) supplemented with 10 g/L of arginine, proline, serine, or tyrosine (i.e., the decarboxylase medium) as reported by Romano et al. [33]. In the modified YPG medium, the concentration of the glucose was reduced to 1 g/L, in comparison to that which is usually used in YPG (20 g/L). This was implemented in order to induce yeasts to use the amino acid added in the medium as their sole energy source. Ten mL/L of a 0.5% solution of pyridoxal 5-phospate was included in the medium (at 0.005%). This was implemented as its presence as a cofactor for the decarboxylation reaction has a strong enhancing effect on the amino acid decarboxylase activity. In addition, 6 mL of a 1% ethanol/water solution of bromocresol purple at a 0.5% final concentration, was utilized as the pH indicator.

The pH was adjusted to 5.2 through HCl 1.0 N and the medium was autoclaved. All the chemicals were purchased from Sigma-Aldrich (Milan, Italy). The strains, grown for 24 h in a modified YPG at 26 °C, were subjected to sterile loops and dissolved in sterile water at a concentration of 10^8^ cells. Five microliters of the cell suspension of each strain were inoculated as “spot” on the surface of the agar plates containing the decarboxylase medium with each amino acid studied; further, the same medium without amino acids was used as control. The plates were incubated at 26 °C for 3 days. At a pH of 5.1 the dye was yellow; however, an increase in pH turned the color to purple. The positivity of the test is indicated by the conversion of the color to purple around the yeast strain spot or, in the case of tyrosine decarboxylation, by the disappearance of tyrosine crystals around the yeast strains that were assayed.

### 2.3. Statistic

Decarboxylation tests were carried out in triplicate. Results were converted into a binary code (1: decarboxylation and 0: no decarboxylation) and used as inputs for the following statistics:

(a) PERMANOVA (permutational analysis of variance), using yeast (*S. cerevisiae*, *Z. bailii*, and *Hanseniaspora* spp.) and amino acids (arginine, proline, serine, and tyrosine) as categorical predictors; moreover, the critical P was set to 0.05. PERMANOVA is a non-parametric approach similar to two-way ANOVA and is useful in order to point out the effect of two or more factors on a dependent variable. The outputs of this statistic are the sum of squares, the mean square (that is the sum of squares standardized to the degree of freedom), the results determined from the Fisher test, and the *p*-value;
(b) Mann–Whitney pairwise comparison test, as the post hoc approach after PERMANOVA, was used in order to point out actual differences amongst batches. Critical P was set to 0.05;
(c) Spearman’s rank order correlation, which is a non-parametric approach in order to highlight the correlation amongst ordinal or ranked variables. Critical P was set to 0.05.
(d) Neighbor joining run on all yeasts, which is a clustering approach in order to group yeasts in phenotypic classes. The similarity index was based on Euclidean distance, while the root was designed through the final branch method.

Statistical analysis was performed through PAST free software ver. 4.2 (Natural History Museum-University of Oslo, Oslo, Norway) [34] and Statistica for Windows, ver. 12.0 (Statsoft, Tulsa, OK, USA).

## 3. Results

The results of the decarboxylation tests, converted into binary codes (1 for a positive test and 0 for a negative test), were treated through the non-parametric approach of PERMANOVA in order to explore the effect of the kind of microorganism, as well as effect on the precursor amino acid and their interaction in regard to the decarboxylation ability. The results of these are shown in Table 2.

Generally, decarboxylation was affected by the character “microorganism” and by the interaction “microorganism × amino acid”, thus highlighting that the amino acid, per se, cannot influence decarboxylation metabolism. However, it should be always understood in combination with the strain due to a possible higher or lower affinity of some yeasts to the four amino acids that were used in this research.

On the other hand, the significance of the predictor microorganism means that some yeasts could possess a higher decarboxylation potential than others.

Conducting PERMANOVA can show the significance of predictors, but it cannot highlight where the difference is, nor which samples are different to each other. In order to achieve this, the output must, instead, be gained through a post hoc comparison test. For this study, the Mann-Whitney test was used and the pairwise comparisons obtained from this are shown in Table 3.

From a practical point of view, comparisons should be understood at two different levels: (i) inside each species/genus in order to explore the possibility that the species/genus possesses a higher or a lower affinity towards an amino acid; (ii) by comparing the results for each amino acid amongst the different yeasts.

*S. cerevisiae* did not show different decarboxylation activities for amino acids, as the rate of positivity to the amino acids varied from 54.88 to 68.29%; further, similar results were found for *Z. bailii* (positivity rate at 54.55% for arginine, proline, and tyrosine, and 64.64% for tyrosine alone). On the other hand, the recovery of the decarboxylation activity for arginine in *Hanseniaspora* spp. was lower (0%) than the values found for the other amino acids (40.91–54.55%), thus showing a difference and a very low affinity for this type of amino acid.

When focusing on the results found for each amino acid in the different yeasts, arginine decarboxylation showed different trends depending on which yeast was utilized. This could be seen with the different rates between *S. cerevisiae*/*Z. bailii* on one hand and *Hanseniaspora* spp. on the other. Differences were also found for serine and tyrosine, as *S. cerevisiae* always showed a higher positivity rate than *Hanseniaspora* spp.

The following research question was on the possible correlation amongst the decarboxylation activities of the different amino acids; thus, positivity rates were analyzed through the Spearman’s test (Table 4).

Serine and tyrosine decarboxylation responses were always related, with correlation coefficients of 0.780 in *S. cerevisiae*, 0.816 for *Z. bailii* and 0.812 in *Hanseniaspora* spp.; moreover, other correlations were also found in the case of *Z. bailii* between serine and proline (0.806), a partial correlation between proline and arginine (0.611), or between arginine and tyrosine (0.612).

**Figure 1:**
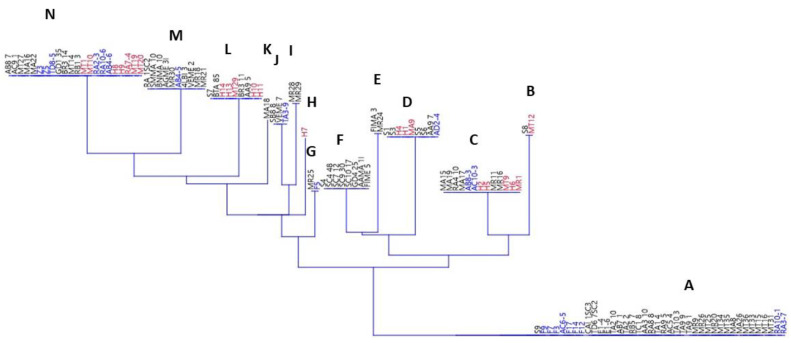
Neighbor joining for yeasts. The responses to amino-acid decarboxylation were used as input data for clustering. The letters on each cluster refer to the phenotypical outputs to the test, as reported in Table S1. Black, *S. cerevisiae*; blue, *Z. bailii*; and red, *Hanseniaspora* spp.

The last statistic was the neighboring joining (shown in Figure 1 and Supplementary Table S1), which was conducted in order to illustrate the eventual phenotypic groups amongst yeasts.

Depending on the response to the decarboxylation activities, 14 different groups were found, ranging from A (positive response to all amino acids) to N (negative response to all amino acids). Group A included 41 strains (9 *Z. bailli* and 32 *S. cerevisiae*), thus showing that ca. 40% of strains for both species were positive to the decarboxylation tests for all amino acids. On the other hand, in group N there were only 22 strains (9 *S. cerevisiae*, 6 *Z. bailii*, and 7 *Hanseniaspora* spp.). The other strains were distributed in the other groups, some of them including only *S. cerevisiae* (for example the groups E, F, I, and K).

## 4. Discussion

Amino acids represent key components for the process of yeast growth as they are strictly related to biomass production, as well as to the synthesis of aroma compounds [35,36,37,38].

Nevertheless, the metabolism of amino acids in yeasts could also pose safety concerns, as it is from amino acid decarboxylation that biogenic amines are produced [39]. However, most of the data available on the metabolism of amino acids are on the *S. cerevisiae* species, while there are few data available on other genera, despite the increasing importance they are gaining in wine microbiology.

Generally, yeasts have a well-defined preference towards amino acids, with some acids usually utilized in the first steps of fermentation, and others utilized after a relatively long time or not utilized at all [20,35]. Recently, Faibairn et al. [35] studied amino acid uptake and evaluated yeast preference through the duration of a lag phase of growth kinetics. That is to say, a lower lag phase means a preference towards an amino acid. For the amino acids tested in this research, we found the lowest values of lag phase (and consequently of fermentation time) was in the case of arginine and serine, whereas the highest value was for tyrosine. The data on proline, on the other hand, are conflicting, as Faibairn et al. [35] reported a lag phase and a yield of biomass production for this amino acid that was not dissimilar to tyrosine, while other authors have reported in the past that proline is not utilized by yeasts [40].

Amino acids have different roles and pathways in the context of yeasts and contribute to different metabolisms. Arginine represents the most important source for yeasts [20]; further, it is involved in both anabolic and catabolic pathways [41].

Serine is a proteinogenic amino acid, is similar to those found in threonine bears, and is also part of an alcohol group. In yeasts, at least in the case of *S. cerevisiae*, whose metabolome has been extensively studied, it plays essential roles (participation in the biosynthesis of purines and pyrimidines, as well as the precursor of glycine, cysteine, and tryptophan) [42].

Tyrosine, as is the case in other aromatic amino acids, can be degraded by yeasts through the Ehrlich pathway in order to produce fusel alcohols, which are important aroma compounds [43].

As such, this research was aimed at carrying out an extensive screening on yeast ability in order to decarboxylate amino acids with different roles in cells and to also gain new data on non-*Saccharomyces* yeasts.

The first output of this research was the high efficiency of *S. cerevisiae* strains, as the decarboxylation yield was >60% for arginine, serine, and tyrosine, and 55% for proline; the high decarboxylation efficiency of *S. cerevisiae* was also supported by the fact that only 8 strains (out of 81 tested in this research, 10% of the total) did not show this trait in, even, the screening test. Nevertheless, many strains (32 out 81) could decarboxylate all amino acids tested.

Amino acid decarboxylation by *S. cerevisiae* could have both positive and negative implications; for example, the decarboxylation of arginine is found in the metabolic pathway and produces certain biogenic amines (i.e., agmatine, spermine, and spermidine) [44], while the decarboxylation of tyrosine and serin leads to the synthesis of aroma compounds [43].

The high recovery of decarboxylation ability for at least one amino acid was also found in *Z. bailii* and *Hanseniaspora* spp., as the strains negative to the tests were, respectively 6 out 21 and 7 out 20; however, a different output for arginine was found in relation to *Hanseniaspora*, as none of the studied strains showed this ability in vitro. This low decarboxylase activity exhibited by *Hanseniaspora* strains is in agreement with the results of Tristezza et al. [45], who found in all isolates an insignificant decarboxylation activity of the amino acids used.

A different profile of amino acids utilization for *Hanseniaspora* was also found by Kemsawasd et al. [46], who reported alanine as the most preferred amino acid for *H. uvarum* and arginine as one of the least preferred. However, it is unclear why *Hanseniaspora* did not decarboxylate arginine as the metabolism of this amino acid is a multi-step reaction with a transamination phase and then a decarboxylation [47]. One of the genes regulating decarboxylation is ARO10 (phenylpyruvate decarboxylases) and it was found highly expressed at the beginning of the stationary phase in *Hanseniaspora*, as well as for the genes involved in the transamination step (ARO8 and ARO9, as well as in aromatic amino acid aminotransferases) [48]. It is most likely that the inability to decarboxylate arginine was related to a lower uptake, which has also been reported by other authors during wine fermentation [49].

Another interesting output was the correlation among decarboxylation abilities of many amino acids (for all yeasts in regard to serine and tyrosine, and serine and proline in regard to *Z. bailii*). In the case of serine and tyrosine we could suggest that amino acids share many steps and intermediates in their metabolic pathways, as well as some gene encodings for these metabolisms (for example serine, tyrosine, and threonine kinase) [50].

Finally, the decarboxylation ability found for proline conflicts with the general model of *S. cerevisiae*’s inability of using it, thus suggesting the needs of further experiments and confirmatory assays.

## 5. Conclusions

Amino acid decarboxylation is a key-metabolism in yeasts, as it is the precursor to the synthesis of aroma compounds and biogenic amines; additionally, the results found in this paper confirmed this statement, as most of the strains were able to decarboxylate at least one amino acid. Moreover, amino acid metabolisms are strongly connected to each other, as also suggested by the high correlation between tyrosine and serine decarboxylation for all yeasts and arginine/proline in *Zygosaccharomyces*, which is most likely related to the sharing of carriers and metabolic pathways.

Finally, *Hanseniaspora* could not decarboxylate arginine, but there are no details in the literature as to why, as the genus has the genes encoding that are required for this metabolism.

Generally, decarboxylation has been underestimated in the selection of yeast strains for wine, but the results of this research have highlighted its importance in regard to the positive or negative implications for wine quality. In addition, this paper has added some new details in terms of differences amongst yeast genera, as well as the interconnections amongst metabolisms.

The experiments in this study were conducted through a screening approach, which allowed the testing of a high number of strains. This is a strength of this research, as generally decarboxylation has only been assessed on a few strains and also only for its negative implications (e.g., the production of biogenic amines), but not for the metabolic implications and the connections amongst different pathways. Future investigations are required in order to focus on the pathways at enzymatic and genetic levels; however, through our data it can be emphasized that the decarboxylase activity that resulted in the strains were dependent, with considerable variability among the species tested, suggesting the use of this qualitative test in the selection program of indigenous strains in order to choose starter cultures possessing potential traits that are also related to health.

Moreover, future perspectives in future studies and other ongoing activities should look to the evaluation of decarboxylation potential under non-optimal conditions, as well as the identification and purification of the enzymes involved in these pathways in order to assess their kinetic parameters.

## Figures and Tables

**Table 1:** List of the yeast strains used in this study: species and strain, origin, isolation year, grape variety, and references.

Species	Strain	Origin	Year	Grape Vine Variety	References
*Saccharomyces* *cerevisiae*	CA1sc3; RA1sc2; TD6-7sc2	Sicily	2004	Nero d’Avola	[28]
	AA3-10; AA9-5; AA9-7; AB7-1; AB8-7; AC5-4; AC9-1; RA4-10; RA8-8; RA9-5; RB1-3; RB5-7; TA1-4; TA2-2; TA2-10; TA9-1; TA9-9; TA10-3; TC1-8	Sicily	2005	Inzolia	[29]
	MA-8; MA-15; MA-16; MA-17; MA-18; MA-19; MA-22; MA-26; MR-9; MR-11; MR-16; MR-18; MR-20; MR-21; MR-24; MR-25; MR-26; MR-28; MR-29; MR-30; MT-14; MT-15; MT-16; MT-25; MT-27; MT-31; MT-33; MT-34; MT-35; MT-36	Sicily	2005	Inzolia	Unpublished data
	S1; S3; S4; S5; S6; S7; S8; S9	Tuscany	2005	Sangiovese	Unpublished data
	BR3-11; BR3-14	Apulia	2007	Primitivo	Unpublished data
	BA-85	Emilia-Romagna	2000	Sangiovese	Unpublished data
	E1-4; E1-6; 4LBI-3; AGMA 1I; FIME 5; FIMA 3; SMMA 10; VEME 2; VEME 7; BNMA 10; AGME 3I	Basilicata	2008	Aglianico del Vulture	[30,31]
	GD1-35; GD4-25	Basilicata	2004	Greco di Basilicata	Unpublished data
	SB8-8; SC4-48; SC7-12; SC9-30; SC10-17	Tuscany	2007	Sangiovese	[32]
*Zygosaccharomyces bailii*	Z3; Z5	Basilicata	2004	Aglianico del Vulture	Unpublished data
	AB8-3; AC10-3; AB4-5; TD8-5; RA2-3; RA10-6; TA3-9; AB4-6; RA10-1; RA3-7; AC6-5; AD2-4	Sicily	2005	Inzolia	Unpublished data
	F3; F5; F7; F9; F12; F14; F17	Liguria	2008	Bosco	Unpublished data
*Hanseniaspora* spp.	MA9; MR-1; MT-1; MT-9; MT-10; MT-12; MT-19; MT-20; MT-29; RA7-4	Sicily	2005	Inzolia	Unpublished data
	H1; H2; H4; H5; H6; H7; H8; H9; H10; H11	Basilicata	2004	Aglianico del Vulture	[7]

**Table 2:** The results of the PERMANOVA run on the data, using a certain kind of amino acids and yeast genus/species as categorical predictors. Critical *p* was set to 0.05.

Source	Sum of Squares	Degree of Freedom	Mean Square	F	*p*
Yeast	6.240	2	3.120	9.056	0.0001
Amino acid	0.728	3	0.704	0.704	0.372
Interaction	−53.315	6	−8.886	−25.789	0.0431
Residual	168.150	498	0.345		
Total	121.80	499			

**Table 3:** Yeasts positive to decarboxylation in recovery percentage (%). Small letters indicate significant differences throughout lines, while capital letters indicate significant differences in a column (Mann-Whitney, *p* < 0.05).

	Arginine	Proline	Serine	Tyrosine
*S. cerevisiae*	67.07 ^a,A^	54.88 ^a,A^	65.85 ^a,A^	68.29 ^a,A^
*Z. bailii*	54.55 ^a,A^	54.55 ^a,A^	54.55 ^a,AB^	64.64 ^a,A^
*Hanseniaspora* spp.	0 ^a,B^	54.55 ^b,A^	40.91 ^b,B^	40.91 ^b,B^

**Table 4:** Spearman’s rank order correlation amongst amino acids in *S. cerevisiae* (**A**); *Z. bailii* (**B**); and *Hanseniaspora* spp. (**C**). Only significant correlations are reported. -: not significant; na: not assessed.; and ns: not significant.

**(A)**				
	**Arginine**	**Proline**	**Serine**	**Tyrosine**
Arginine		-	0.316	0.303
Proline	ns		0.484	0.435
Serine	0.316	0.484		0.780
Tyrosine	0.303	0.435	0.780	
**(B)**				
	**Arginine**	**Proline**	**Serine**	**Tyrosine**
Arginine		0.611	-	0.612
Proline	0.611		0.806	0.816
Serine	-	0.806		0.816
Tyrosine	0.612	0.816	0.816	
**(C)**				
	**Arginine**	**Proline**	**Serine**	**Tyrosine**
Arginine		na	na	na
Proline	na		-	-
Serine	na	-		0.812
Tyrosine	na	na	0.812	

## Data Availability

Data is contained within the article or Supplementary Material.

## Supplementary Materials

The following supporting information can be downloaded at: https://www.mdpi.com/article/10.3390/foods11223587/s1. Table S1: Phenotypic groups based on the responses to the decarboxylation of amino acids.
