# Supplementary material for: Screening of Saccharomyces and Non-Saccharomyces Wine Yeasts for Their Decarboxylase Activity of Amino Acids
Source: Foods. 2022 Nov 11;11(22):3587. doi: 10.3390/foods11223587 (PMC9689846; doi:10.3390/foods11223587)
Supplement: Supplementary file 1 [file foods-11-03587-s001.zip › foods-1998493-supplementary.pdf]

Supplementary material

Table S1: Phenotypic groups based on the responses to the decarboxylation of amino acids

	**Arginine**	**Proline**	**Serine**	**Tyrosine**
A	1	1	1	1
B	0	1	1	0
C	0	1	1	1
D	0	0	1	1
E	1	0	1	0
F	1	0	1	1
G	1	1	0	1
H	0	1	0	1
I	0	0	0	1
J	1	0	0	1
K	1	1	0	0
L	0	1	0	0
M	1	0	0	0
N	0	0	0	0
